# Evidence and consequences of academic drift in the field of dental research: A bibliometric analysis 2000–2015

**DOI:** 10.1038/s41405-022-00093-w

**Published:** 2022-01-17

**Authors:** Puck van der Wouden, Geert van der Heijden, Hagay Shemesh, Peter van den Besselaar

**Affiliations:** 1 Oral Public Health Department, ACTA, Amsterdam, the Netherlands; 2 Department of endodontology, ACTA, Amsterdam, the Netherlands; 3 Vrije Universiteit Amsterdam, the Netherlands & Deutsche Zentrum für Hochschul- und Wissenschaftsforschung (DZHW), Berlin, Germany

**Keywords:** Extended skills training in dentistry, Dentistry

## Abstract

The mission of academic excellence has resulted in a science system that incentivises publications within high impact, often basic science journals, and less in application-oriented journals. For the dental research field this so-called academic drift can result in a research portfolio that moves away from research that serves dental healthcare. Therefore, we examined if and how academic drift has changed the dental research field. Web of Science data were used to develop a network map for dental research containing journal clusters that show similar citation behavior. From the year 2000 up to 2015, we explored the intensity of knowledge exchange between the different clusters through citation relations. Next, we analyzed changes in research focus of dental research institutes in seven countries, in *dental research, clinical medicine research, basic science, public health research* and *other fields*. Within the citation network, 85.5% of all references in dental journals concern references to other dental journals. The knowledge contribution of non-dental research fields to dental research was limited during the studied period. At the same time, the share of output of dental research institutes in dental research has declined. The research activity of the dental research institutes increased mainly in basic science while the knowledge input from basic science into dental research did not increase. Our findings suggest that the dental research portfolio is influenced by academic drift. This academic drift has increased the disbalance towards basic science, and presents a challenge for the scientific progress in dental healthcare services.

## Introduction

The science system functions as a reputation system, and researchers are inclined to choose research lines and publication strategies that may boost their reputation, which mainly is achieved through recognized contributions to science [1]. Thereby, researchers are amenable to choose research themes and publication strategies that may boost their reputation. The research systems of advanced countries stimulate the production of large volumes of high-quality publications in top journals [2, 3]. For many research institutes, academic excellence and the science frontier is pivotal for their mission, their research policy and research portfolio [4]. Moreover, in the last decades, academic excellence has been a dominating feature of the performance evaluation of researchers and research groups. At the same time, the emphasis on academic excellence has been criticised, as it has resulted in a science system that is driven by incentives aiming at high citations and impact factor scores. It thereby disregards a major goal of science which is to respond to the needs and challenges of society, by creating relevant knowledge that brings benefit to society [5–8].

In dental research, according to the Dutch Health Council, the focus on ‘excellence’ has resulted in a changing dental research portfolio. That is, in 2010 already about half of the total output of the three Dutch academic dental institutions concerned research in the basic sciences - as opposed to applied research [9]. This seems to indicate that ‘academic drift’ occurs in dental research, notably, “*the process whereby knowledge which is intended to be useful gradually loses close ties to practice while becoming more tightly integrated with one or another body of scientific knowledge” (p. 413)* [10]. Drift in this sense has been a common phenomenon in many domains of science, including agriculture, engineering, medicine, education and management. The emphasis on academic excellence has been translated in the medical (including dental) domains in an emphasis on publications in journals with high impact factors, the so-called ‘top journals’. This most likely has affected research interests and the choice of research topics, as the majority of publications in those ‘top journals’ concern the basic sciences [11, 12]. Applied sciences and in particular research addressing local or regional needs and practical challenges are considered outside of what is conceived as the frontier of science. Therefore, as a result of ‘academic drift’, a tendency to publish in high impact international basic research journals, and less in application oriented - often local or national – journals can be expected. As a consequence, the dental research field may partly move away from the more practical questions that emerge from everyday dental care practices [9].

The academic drift and the reputation system both harbor the risk of pursuing exclusively progress in science, thereby neglecting innovation and progress in society as a universal target. However, more recently the societal relevance of research has gained a more prominent place in evaluation and funding systems, which has resulted in a new paradigm of ‘translational science’ [13–15]. Because of the slow pace of the implementation of this paradigm shift, it remains to be seen whether it will reverse the impact of the reputation system and its impact in terms of the imbalances due to academic drift.

The purpose of this study is to provide insight in the occurrence of academic drift in the dental research field and whether and how this has impacted this field. Therefore, we address the following questions:

1. Is the balance between applied and basic research in the dental research field shifting away from applied research, which is oriented to dental care practice?
2. What is the share of non-dental research within the portfolios of the dental research institutes, and what is the balance between dental and non-dental research?
3. Does non-dental research provide pertinent knowledge which is relevant for and of benefit for dental research?

In order to answer these questions, we use bibliometric methods to analyze trends in the dental research field from the year 2000 up to 2015 at a global level and for several important research countries. We use three approaches to operationalize academic drift.

As a first indicator of academic drift we use the change in research focus of *dental research institutes*. For this, we analyze whether research activities of dental research institutes in non-dental (and more basic) research fields increase at the expense of dental research. Therefore, we analyze changes in volumes of publications of dental research institutes in the dental and non-dental research fields.

Secondly, non-dental research may have relevance for dental research (basic science may inform applied science). Therefore, as a second indicator of academic drift, we analyze which non-dental research fields function as knowledge suppliers for dental research.

Thirdly, while many dental journals have an international orientation, readership and authorship, national journals may serve more locally and nationally oriented authors and readers, including dental practitioners. Hence, as third indicator of academic drift, we address the role of national journals, and study the changes in the volume of publications in the local dental journals.

## Material & methods

### How to analyze change in research fields?

The dynamics of science can be studied best at the macro level of the communication of research findings (publications), and not at the micro level of research activities [16]. Within most research disciplines, publication in scientific journals is the main vehicle of communication. This enabled us to study the change in a research domain by analyzing the dynamics of the journal structure [17]. In our study we initially define dental research as publications categorized in the Web of Science (WoS) category *Dentistry, Oral Surgery & Medicine*. WoS has a wide coverage of science and journals are categorized in WoS categories that correspond to research fields. Most importantly WoS provides citation information, which allows us to study science dynamics. Then we derive the main dental research journals from the WoS category, and define dental research in terms of journal clusters, which enables to map the structure of and knowledge streams within the dental research field and its environment. To identify research done within the dental research institutes we use the classification based on WoS categories, as this enables to analyze the changes in the topical focus of those institutes in relation to academic drift.

### The changing place of dentistry in the scientific landscape

To analyze the structure and change of the dental research field from 2000 up to 2015, we first determined what the dental research field comprises. Therefore, we used the set of journals classified in the WoS category *Dentistry, Oral Surgery & Medicine* and indexed in the InCites Citation Reports with a Journal Impact Factor. Hereafter, we will refer to this set of dental journals as the “*core-set*”.

In order to understand the changes in the research field of dentistry, we mapped the place of dentistry in the scientific landscape for the beginning, the middle and the end of the studied period: 2000, 2008, and 2015. This was done in the following way [17]. For each of these years, we selected the 27 journals with the highest impact factor from the *core-set*. (Annex A) These journals are related to each other and to other journals through citations. We then identified all journals that either cite (at least) one of the selected journals or are cited by (at least) one of the selected journals. These related journals are dental and non-dental journals as relevant research is not necessarily limited to exclusively dental journals. We restricted our analyses to related journals above a threshold of 0.5% [17] of the total number of citation relations with at least one of the selected journals (either citing or being cited). The lower numbers of citations are considered ‘noise’.

Factor analysis was used to identify clusters of journals that have similar citation behavior in the network. Journals with similar citation behavior belong to the same research field or subfield. As we started with our *core-set*, the network map is expected to show various dental research subfields, as well as other research fields that either are cited by these dental journals, or cite these dental journals themselves. The names of dental clusters were based on similarities in the journal titles, and corroborated by two authors who are field specialists (PvdW and HS). The names of non-dental journal clusters were based on the WoS category to which most journals of a cluster were assigned.

The Netdraw tool (Borgatti, S.P., 2002. NetDraw Software for Network Visualization. Analytic Technologies: Lexington, KY.), which was designed for visualizing (social) network relations, and is included in the social network analysis tool Ucinet (Borgatti, S.P., Everett, M.G. and Freeman, L.C. 2002. Ucinet for Windows: Software for Social Network Analysis. Harvard, MA: Analytic Technologies), which was used to visualize the network. The visualization was done using the Graph theoretical layout with a threshold of 0.3 for relations between the nodes.

The network map shows the larger disciplinary landscape of and around dental research. This enables us to explore the structure and intensity of knowledge exchange (so-called knowledge streams) between the different clusters of journals using citation relations. The strength of the knowledge stream is determined by the number of times journals from one cluster are cited by journals from another cluster. Changes in citing behavior are an indicator of cognitive change [18], therefore we compare citation relations between journal clusters for the three years.

### Analyzing the research focus of the dental research institutes

We compared the scientific output of identified dental research institutes between several countries in the WoS category *Dentistry, Oral Surgery & Medicine* with the output in other WoS categories. We included seven countries that have a well-developed research system and have contributed significantly to trends in the dental research field. Thus, we focused on countries that perform well in terms of quality and quantity of publications. In Annex-B it is described how these seven countries were selected. We expected dental research institutes to be the main source of publications in the dental research field, and therefore used the publications affiliated to dental research institutes as the source of publications for the selected countries. We tested if the dental research institutes are indeed the main source of dental publications by estimating the contribution of the dental research institutes to the total output in WoS category *Dentistry, Oral Surgery & Medicine*. The use of dental research institutes allows to determine ratios between dental and non-dental publications.

The dental research institutes, notably research institutes with one of the main dentistry concepts in their name, were identified using a dedicated query (see Box 1). This query exploited two address fields within a WoS record. The field tag AD for address was used to identify relevant research institutes with one of the main dentistry concepts in their institute name. The field tag SG for Suborganization was used to identify relevant research suborganizations with one of the main dentistry concepts in their name. This query was repeated per country. Publications affiliated with dental research institutes from multiple included countries were attributed to all included countries.

We then analyzed the changes in the publication activities of dental research institutes (aggregated on country level). First, the volume of research output by the dental research institutes in the *core-set* journals and the volume of total research output was determined. We analyzed growth in both volumes per country and worldwide. We calculated the share of research output in the *core-set* of the total research output for the periods 1998–2000 and 2014–2015. As research output of especially smaller countries may vary over the years, we use output over multiple years to compare the countries. Over time, overall the output has increased considerably. Therefore, we use three consecutive years (1998–2000) in the earlier period and two consecutive years (2014–2015) in the later period.

Box 1 WoS queries for dental research institutes Per country:

(AD = ((dent* NEAR/15 Country) OR (Cario* NEAR/15 Country) OR (Endodont* NEAR/15 Country) OR (Pedodont* NEAR/15 Country) OR (Periodont* NEAR/15 Country) OR (“Oral Biochemistry” NEAR/15 Country) OR (“Oral Cell Biology” NEAR/15 Country) OR (Implantol* NEAR/15 Country) OR (Prosthod* NEAR/15 Country) OR (“Oral Radiology” NEAR/15 Country) OR (“Oral Kinesiology” NEAR/15 Country) OR (“Oral Medicine” NEAR/15 Country) OR (Orthodont* NEAR/15 Country) OR (Maxillofac* NEAR/15 Country) OR (orofac* NEAR/15 Country)))

AND

(SG = (dent* OR Cario* OR Endodont* OR Pedodont* OR Periodont* OR “Oral Biochemistry” OR “Oral Cell Biology” OR Implantol* OR Prosthod* OR “Oral Radiology” OR “Oral Kinesiology” OR “Oral Medicine” OR Orthodont* OR Maxillofac* OR orofac*)) *AND* **DOCUMENT TYPES:** (Article OR Review)

World-wide

(AD = ((dent*) OR (Cario*) OR (Endodont*) OR (Pedodont*) OR (Periodont*) OR (“Oral Biochemistry”) OR (“Oral Cell Biology”) OR (Implantol*) OR (Prosthod*) OR (“Oral Radiology”) OR (“Oral Kinesiology”) OR (“Oral Medicine”) OR (Orthodont*) OR (Maxillofac*) OR (orofac*))) *AND* **DOCUMENT TYPES:** (Article OR Review)

### Output by dental research institutes in the wider landscape

We further analyzed in more detail to which WoS categories publications from dental research institutes were attributed. For each country, we calculated the share of publications by the dental research institutes per WoS category for the first period (1998–2000) and for the last period (2014–2015) to analyze (pattern of) changes in the importance of the research fields over time.

### The changing focus on local journals

Based on the assumption that applied research has a tendency to be published in journals with a national or local orientation, we calculated the share of total output in local journals as indicator for applied research within the dental research field [19–24]. We considered journals related to international societies, like the *Journal of Dental Research* or the *International Dental Journal*, or targeting a particular dental specialism, like *Caries Research* or the *Journal of Clinical Periodontology*, to have an international orientation, when published in the English language. We categorized as local journals those either including a country name in their title, like *Swedish Journal of Dentistry*, or when published in the national language. One may argue however that journals including British, American, Australian, or New Zealand in their journal titles are not exclusively local because they are published in the English language. Therefore, we calculated the correlation between the countries’ share in the *core-set* and the countries’ share in journals that might be identified as local (based on the journal title). If this correlation was less than 0.5 the journal was identified as local. The journals in Annex-C are strongly dominated by publications from one or two countries, and were therefore classified as local.

Then we analyzed, per country, the share of total output of dental research institutes in the local journals, which may help to understand the national differences in the (application) orientation of dental research.

## Results

### The place of dentistry in the scientific landscape

The resulting journal network for the year 2008 consists of about 250 journals, of which we used 187. We excluded 63 journals, as these are in the WoS database only as cited items. The network analysis of the 187 journals results in 31 clusters, each representing a research field or subfield. Some clusters obviously represent a dental research subfield, like dental materials and dental public health (see Table 1: clusters 0, 2, 4, 5, 8, 10, 15, 16, 22) while other clusters represent a research field that is related to dentistry primarily through citation relations, like oncology and pain.

**Table 1:** Clusters within the dental journal network (2008).

	Journal cluster^a^				
0	General dentistry	12	Clinical microbiology	24	Biomechanics
1	Oncology	13	Pain	25	Laser
2	Operative dentistry & materials	14	Otorhinolaryngology	26	Public health USA
3	Public health/general medicine	15	Endodontology	27	Genetics
4	Implantology	16	Periodontology	28	Chemistry
5	Community dentistry	17	Pediatrics	29	Geriatrics
6	Plastic surgery	18	Neuroscience	30	Quality of life
7	Biochemistry	19	Immunology	31	Forensic science
8	Orthodontics	20	General medicine	32	Radiation
9	Microbiology	21	Anatomy	33	Anthropology
10	Oral surgery	22	TMD	34	Medical devices
11	Biomaterials	23	Kinesiology		

^a^Annex-D displays how the journals are distributed over these clusters

**Fig. 1:**
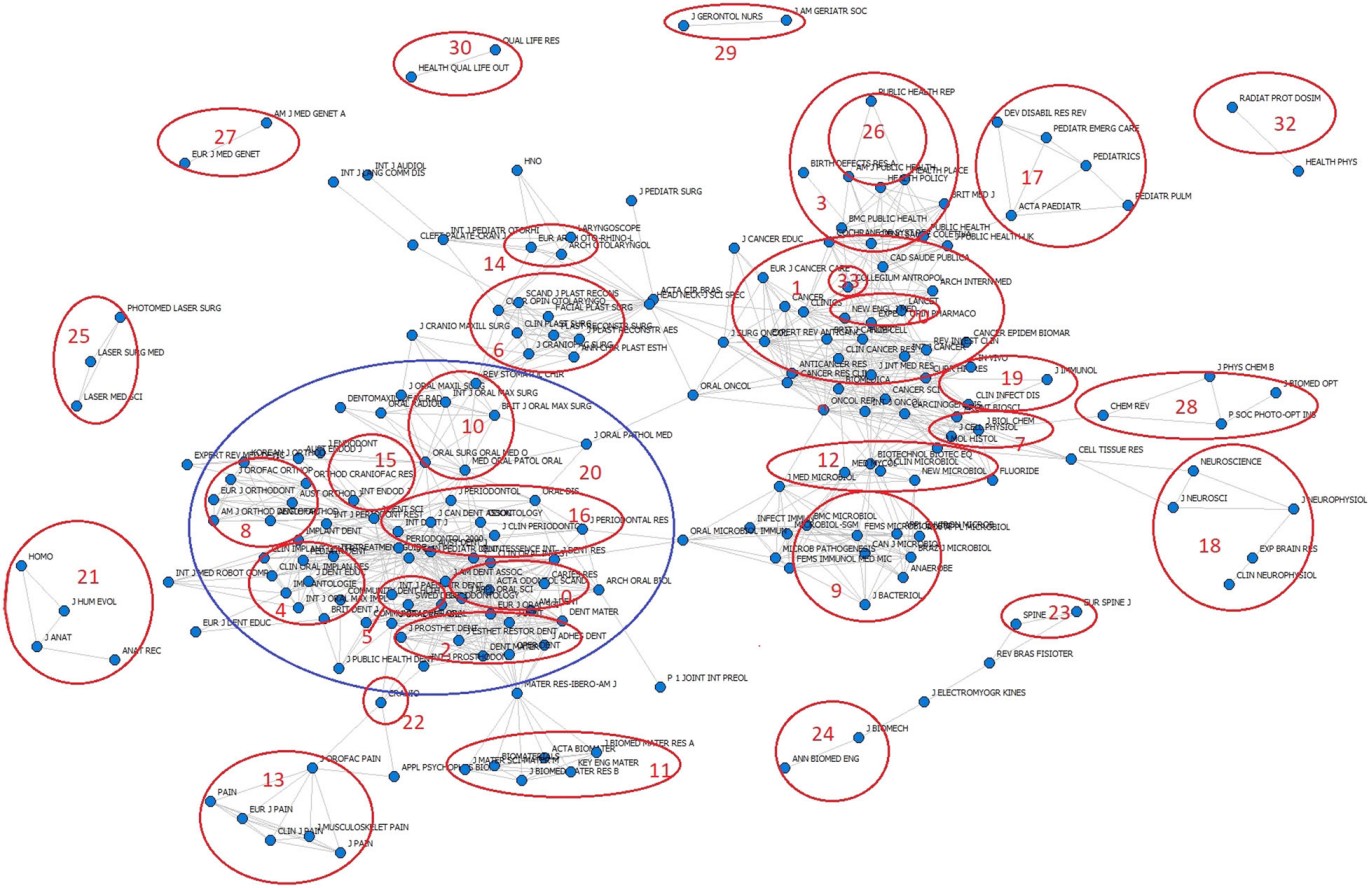
Map of the journal network of the dental research field (2008), with the main fields indicated. The network was produced with *Netdraw*, with the *Graph theoretical layout* with a threshold of 0.3. The circles on the map were added manually.

Figure 1 shows the map of dental research and the main neighboring fields. Each node represents a journal and all journal clusters are indicated on the map by a circle. In the large blue circle, the journal clusters representing dental research subfields are found. In Annex-D, an overview of all clusters including the journals belonging to them is found.

Quite a few of the non-dental clusters found in fig. 1 [Clusters 7, 9, 11, 13, 18, 19, 21, 24, 27, 28, 33] seem to represent basic research that may be used within dental research. The remaining clinical [Clusters 1, 6, 12, 14, 17, 20, 23, 29], public health related [Clusters 3, 29, 30], instrumental, and other [Clusters 25, 31, 32, 34] fields may be used within dental research, or may be using dental research results. This can be visualized using a map that represents the knowledge streams between the clusters.

**Fig. 2:**
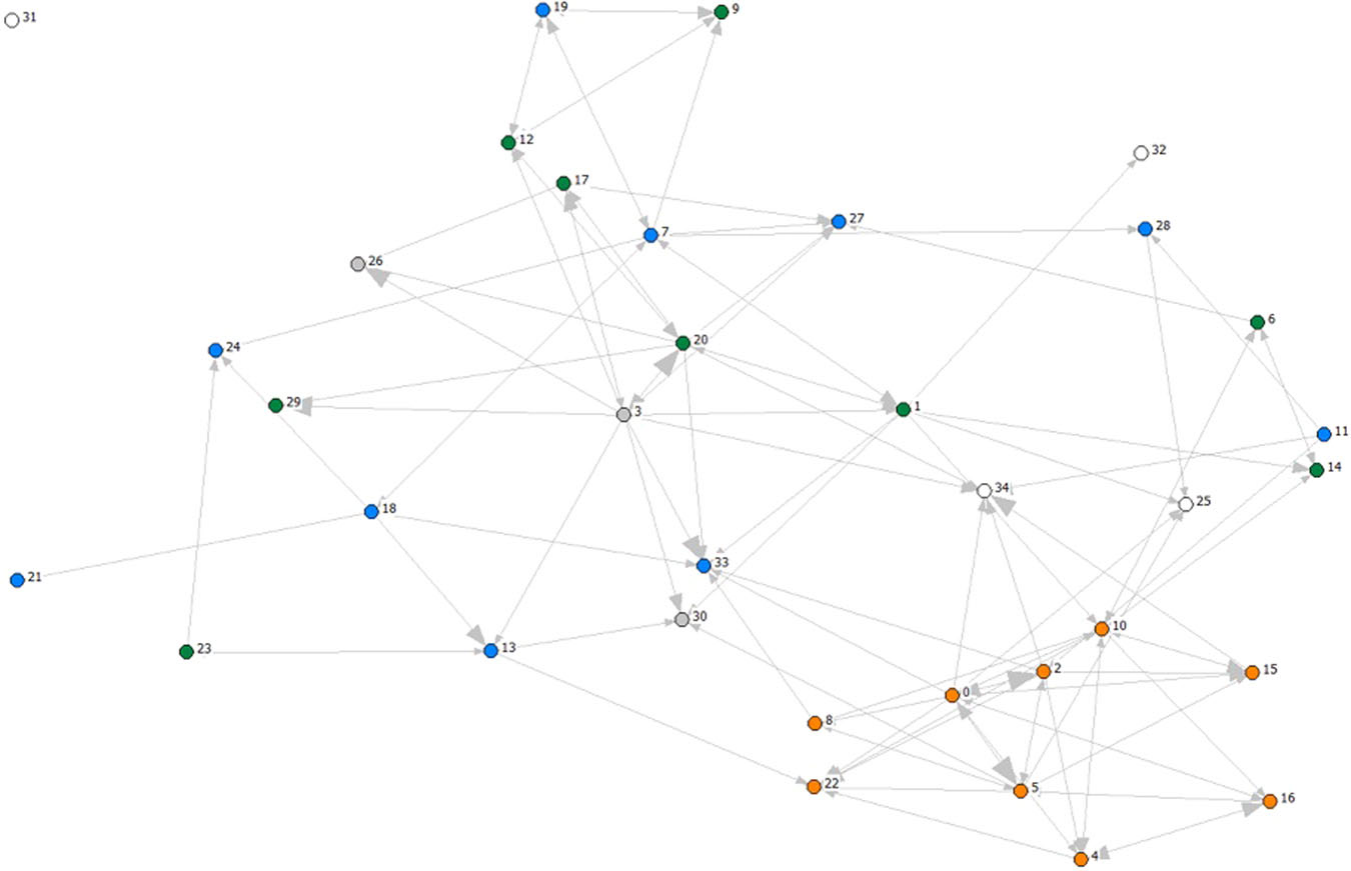
The knowledge streams around dental research 2008. The numbers refer to the research fields in Table 1. The size of the arrows head indicates the strength of the knowledge streams. The network was produced with *Netdraw*, with the *Graph theoretical layout*. We only visualize the stronger links (larger than 3% of all references of the journals in a field). Node colors: Orange = dental research clusters; Green = clinical medicine research clusters; Blue = basic science clusters; Grey = public health research clusters; White = other.

Figure 2 shows the knowledge streams between the main research fields for 2008. The map consists of the observed journal clusters, symbolized by a colored node, and these represent a research field. The citation relations between the fields are represented by the arrows between the clusters, and the direction of the arrow indicates the direction that the knowledge streams. The more citations of publications from journals in the cluster the arrow are pointing at, the thicker the point of the arrow is. We visualized only the stronger links. The streams can go in both directions, but that is not necessarily the case. In Fig. 2, the arrow from public health to community dentistry indicates that the community dentistry journals are citing the Public health journals but are hardly cited by public health journals.

The dental clusters (orange nodes) are concentrated on the right part of the chart (Fig. 2). Direct knowledge streams from non-dental clusters to dental clusters are limited. The following knowledge streams (represented by an arrow) to a dental cluster can be distinguished: The biomaterials cluster contributes knowledge to the dental materials and implantology clusters, the public health cluster contributes knowledge to community dentistry cluster, while the plastic surgery and oncology clusters contribute knowledge to the oral surgery cluster.

All other clusters at best indirectly contribute knowledge to the dental research clusters. For clarity, we aggregated the knowledge streams for dental and non-dental clusters. Within the network, 85.5% of all references in dental journals concern citations of other dental journals, whereas 14.5% of the references in dental journals concern citations of the non-dental journals in the network. This shows that the non-dental fields contribute limited knowledge to dental research, indicating an inward orientation of dental research.

### Comparing 2000, 2008 and 2015

The knowledge streams for the years 2000 and 2015 were analyzed as well. The network map of the scientific landscape of dental research changes between the years, but the main research fields and subfields are found in all three network maps (Table 2).

**Table 2:** Comparing the cluster structure over 2000, 2008, and 2015.

	2000	2008	2015
**Dental research**			
General Dentistry	x	x	x
Operative Dentistry / Dental Materials		x	x
Oral rehabilitation	x		
Endodontics	x	x	x
Implantology	x	x	x
Oral and Maxillofacial surgery	x	x	x
Community dentistry	x	x	x
Dental education			x
Oral oncology			x
TMD		x	x
Orthodontics	x	x	x
Periodontology	x	x	x
**Public health**			
Public health (USA)		x	x
Public health/General medicine		x	
Quality of life research		x	x
**Clinical medicine**			
General medicine	x	x	x
Clinical microbiology	x	x	x
Oncology	x	x	x
Dermatology	x		x
Pediatrics		x	x
Neurosurgery	x		
Plastic surgery	x	x	x
Ophthalmology	x		
Orthopedics	x		
Geriatrics		x	
Otorhinolaryngology	x	x	x
**Basic science**			
Neurosciences		x	
Virology	x		in interdisciplinary
Microbiology		X	x
Immunology	x	X	x
Anatomy	x	X	scattered
Biochemistry	x	X	x
Chemistry		X	
Biomaterials	x	X	x
Material sciences	x		x
Bone	x	Incl. biomechanics	x
Kinesiology		X	
Genetics	x	X	x
Anthropology	x	One journal	x
Pain	x	X	
Pathology	x		
Speech	x		x
Stem Cell transplantation	x		
Develop biology			x
Interdisciplinary			x
**Other**			
Laser	x	X	x
Forensics		X	x
Radiology	x	X	
Medical devices		One journal	x

We aggregated the knowledge streams for the three years into streams between the five groups as found in Fig. 2: *dental research, clinical medicine research, basic science, public health research* and *other*. Table 3 shows the knowledge streams towards dental research clusters in the three years. In 2000, 80% of the references in the dental journals refer to other dental journals. The remaining 20% references were mainly to clinical medicine journals (12%), and basic science journals (8%). In 2008, the share of references to other dental journals was even higher (85%), while in 2015, the pattern was about the same as in 2000. Based on these findings we conclude that the dental research field mainly depends on knowledge produced within the dental research field. The knowledge streams from basic science into dental research have not increased much over the fifteen years period.

**Table 3:** Knowledge streams to dental research clusters, per group.

	Dental research 2000	Dental research 2008	Dental research 2015
Dental research	80%	85%	79%
Clinical research	11%	7%	10%
Basic science	8%	6%	9%
Public Health research		1.4%	0.9%
Other	0.7%	0.4%	0.6%

### The research focus of the dental research institutes

In the previous section, we reported patterns in the dental research field at the global science level. In this section, we focus on the changing research portfolio of the dental research institutes in the seven selected countries (based on the largest dental research output in high impact factor dental journals) namely: USA, England, Germany, Italy, The Netherlands, Sweden, Switzerland.

Table 4 shows the dental output published by *non-dental research institutes* for the selected countries in the WoS category *Dentistry, Oral Surgery & Medicine*. In the first period, the share of total output by non-dental researchers in the *core-set* is between 10% and 23% and it declines to between 3% and 15%. Clearly, dental research institutes produced the overwhelming part of dental research output, and we consider the output of those institutes to be representative for the dental research field. Hence, we restricted the next analysis to these dental research institutes.

**Table 4:** Share of total output of non-dental research institutes within WoS category *Dentistry, Oral Surgery & Medicine*.

	1998–2000	2014–2015
Sweden	23%	15%
Switzerland	11%	9%
Germany	17%	7%
England	10%	6%
USA	13%	6%
Italy	10%	5%
The Netherlands	13%	3%
Average	14%	7%

Publications by dental research institutes are not restricted to dental journals. We calculated the distribution of the publications over the WoS categories for the dental institutes from the seven countries. Based on Fig. 2, WoS categories were grouped into *dental research, clinical medicine research, basic science, public health research* and *other*. Annex-E displays to which group each WoS category was assigned. As displayed in Table 5, the share of output in the *dental research* group is still the largest, but has declined from 61% to a 43%. Shares of output in all non-dental groups increased, with the share of *basic science* increasing from 33% in ’98-’00 to 40% in ’14-’15, and the group *other* increased from 5% to 8% over this period. The increase in this group is attributed to the WoS category *Multidisciplinary sciences* covering journals like Science, Nature and PNAS, journals that can be included under basic science. The more fine-grained changes at the level of individual WoS categories are found in Annex-E.

**Table 5:** Distribution of all output from dental research institutes, seven countries, over five groups 1998–2015.

Group^a^	1998–2000	2014–2015
Dental research	61%	43%
Basic science	33%	40%
Clinical research	19%	20%
Other	5%	8%
Public Health research	2%	4%

^a^The total is higher than 100% as some journals are classified in more than one group

Table 6 shows an overview of the growth of research volume for dental research institutes worldwide and for the seven countries. We determined growth in output in the *core-set* as well as the growth in the total output.

**Table 6:** Changes in publication patterns, output of dental research institutes aggregated per country, 1998–2015^a^.

	Growth output in *core-set* 1998–2015	Growth in total output 1998–2015	*Core-set*/Total 98–00	*Core-set*/Total 14–15
World	247	330	52%	39%
Italy	441	624	60%	43%
Switzerland	441	543	70%	57%
Germany	347	440	61%	48%
The Netherlands	209	356	64%	38%
England	129	224	45%	26%
USA	158	203	49%	38%
Sweden	125	180	74%	52%

^a^Index: 1998 = 100

During the studied period, the growth of publications in the *core-set* varied between 25% (Sweden) and 341% (Italy, Switzerland). In all countries, the increase in total publications (dental publications and publications outside of the dental journals, authored by dental research institutes) is larger than the increase in the number of dental research publications. This means that dental institutes publish relatively more in non-dental journals in the latter period. As a result, the share of dental output within the total output declines. In most countries, that percentage has fallen under 50%, and in England it is only 26%.

Based on the findings presented in Table 3 and Table 5, we conclude that the research activity of the dental research institutes diversified strongly, leading to an increased activity in basic research at the expense of dental research: the share of dental output declined. However, the knowledge streams from the basic science fields to dental research have not increased over the period. This suggests that dental research is not so much becoming more science-based, but that the research activities of dental research institutes are showing academic drift.

### The role of local journals

In the introduction we distinguished two pressures on the science system: (i) to increase excellence, resulting into academic drift, and (ii) to increase societal relevance. Above we showed that activities of dental research institutes increasingly move into non-dental research fields, where publications may have more impact. Next, we compared the role of local dental journals over time between the seven countries, as that may be used to illustrate the role of societal (dental care-related) relevance. In national research systems that demand knowledge transfer to ‘end-users’, researchers may be more inclined to publish also in local journals that may reach practitioners. Please note, if a German researcher publishes in a Swedish national journal, this is also counted as ‘local’ for the German researcher, as we assume that the type of publication, and not the country, is the pivotal difference between international and local journals.

To investigate the focus on societal relevance, we identified the local dental journals (Annex-C), and calculated for the dental research institutes in each of the countries the number of publications they have in these local journals. For each country, we then calculated the share of local publications of the total output. Table 7 shows the results for 2000 and 2015. The shares of local publications are very different between the countries in 2000: from 1% in Italy to 18% in Sweden. In 2015 these differences are much smaller: from 4% in the USA to 12% in Sweden. To quantify the decrease in differences, we calculated the coefficient of variance for 2000 and 2015 for the seven countries. The differences between the countries decline (the coefficient of variation decreases from 0.92 in 2000 to 0.30 in 2015). A possible explanation for this convergence is that in countries where the share of publications in local journals was high, the pressure on excellence has resulted in a declined focus on local publications. On the other hand, in countries where the share of publications in local journals was low, the increasing pressure on the science system for societal relevance may have caused an increase in local journal publications.

**Table 7:** Publications of dental research institutes aggregated per country in local journals, as share of all publications.

	2000 Share in local	2015 Share in local
Sweden	18%	12%
England	16%	10%
Italy	1%	9%
Germany	5%	7%
Netherlands	2%	7%
Switzerland	3%	7%
USA	4%	4%
Coefficient of Variance	0,92	0,30

Take for example the Netherlands, where both pressures exist [25]. We see on the one hand an increase in publications in local journals (from 3% to 7% between 2000 and 2015), suggesting responsiveness of the research system to societal demand. On the other hand, the increase in publications in non-dental journals (from 36% to 62% between 2000 and 2015 – Table 6) suggests an academic drift.

## Discussion

We reported an evaluation of the research dynamics within the dental research field through different approaches, and determined the place of dental research within the scientific landscape. Our analyses showed that dental journals have a distinct position on the journal network map, and the citation relations between the different dental research fields are much stronger than those with non-dental research fields. In addition, we have identified the rather limited knowledge streams from non-dental clusters to dental journal clusters, and these streams have not increased over time. These findings indicate that dental research constitutes a mono-disciplinary, and very likely even an insular research field [26].

Due to academic drift the dental research portfolio has changed. The share of the dental research activities serving scientific progress in the basic sciences has grown the most in absolute and relative terms. Consequently, the balance in the dental research portfolio has further shifted towards basic research.

As shown, dental research institutes provide the overwhelming and increasing part of dental research. The focus of dental research institutes, however, has strongly shifted towards publications in non-dental research fields. For 5 of the 7 countries included in our analysis, less than half of the output of dental research institutes can be classified as research published in the *core-set* (Table 6). Nowadays, dental research institutes particularly publish in basic sciences journals and clinical medicine journals, and the research activities (in terms of publications output) of dental research institutes show a drift away from dental research. At the same time, the knowledge streams from non-dental research to dental research are limited and rather stable. This suggests that the relevance of this non-dental research for dental research and practice may be limited. However, this raises the question whether dental research benefits from non-dental research through other mechanisms than citation relations, for example through research collaboration, through informal contacts, or through the use of medical instrumentalities [27, 28].

Through three different approaches our study showed how the dental research field is changing. The main contributors to dental research – the dental research institutes – shift their focus to non-dental research. However, the contribution of non-dental research to dental research seems to remain limited. This is a strong indicator for the occurrence and impact of academic drift in the dental research field.

How do our findings relate to those of others? A literature search (date 12^th^ march 2020) resulted in 52 publications in WoS category *Dentistry, Oral Surgery & Medicine* that include a bibliometric analysis. Most of these publications focus on highly cited articles or on bibliometric indicators for a specific dental research field [29–31], a specific journal [32, 33] or a specific country [34, 35]. Only two publications approached the entire dental research field. Gil-Montoya et al. (2006) used a cross-sectional study design to quantitatively and qualitatively compare contributions from different countries to the dental research field [36]. They concluded that a substantial part of the activities in the dental research field come from a limited number of countries. Pulgar et al. (2013) analyzed dental research including dental publications outside of WoS category *Dentistry, Oral Surgery & Medicine* per country using a topic search strategy [37]. They found, similar to our findings, an increase of dental publications especially in WoS categories covering basic science.

To our knowledge only Skvoretz et al. (2016) analyzed in a cross-sectional study the knowledge exchange – in terms of citation patterns - between the dental research field and one non-dental research field, namely prenatal research [38]. A keyword search was used to identify the dental and prenatal publications. Similar as in our study, they report that dental research (as well as prenatal research) shows ‘inbreeding’ tendencies in terms of citation behavior.

In our study we did not limit our analysis to knowledge exchange between a particular non-dental and dental field, but we used the publication output of dental research institutes, as this level of aggregation allowed us to not only move beyond analysis of citation relations with non-dental fields, but also to identify in which non-dental research fields dental research institutes publish: especially in surgery, in biomedical & tissue engineering, and in biomaterials (Annex-E).

While Haslam et al. (2011) found in general similar sparse citation relations between psychiatry and clinical psychology within the field of mental health research, in future research it remains to be shown whether our findings are typical for the dental research field or indicate a more general pattern in biomedical research or even in science [26].

A limitation of our study is the possible misclassification, notably publications from dental research institutes which cover dental research topics may have been classified as non-dental. Pulgar et al. reported that in the period of 2006–2008 approximately 15% of all dental publications (identified through a keyword search for dental topics) were published in a non-dental WoS category [37]. Also, the opposite misclassification is possible, as publications within WoS category *Dentistry, Oral Surgery & Medicine* may cover basic science which eventually may not be related to dental research. Since we did not use publication level for our analysis, it remains unclear how much publications were misclassified due to WoS categorization. However, dental research publications from dental research institutes in basic science journals would have been reflected in the gross-group citation patterns. As the share of publications of dental research institutes within non-dental journals has increased over time, the citation relations between non-dental and dental research have not increased. Therefore, we are convinced that a potential misclassification might only have limited impact on our findings and that our method is adequate for analyzing research dynamics of academic drift.

Furthermore, the share of publications from dental institutes in non-dental journals is much larger (57%) than the 15% through a topic search strategy found by Pulgar et al. which strengthens our findings that the largest part of publications in non-dental journals are classified as non-dental research correctly.

One might argue that the findings about academic drift and pressures towards societal relevance may be the effect of the selection of the countries. However, trends in a research field are foremost determined by countries with advanced research systems that contribute large volumes of publications, which justifies our selection of countries [39].

As expected, dental research is for the largest part embedded within dental research institutes. A shift in the focus of these research institutes to other research fields holds important implications for the dental research field. The academic drift towards more basic science consequently has an effect on basic and applied dental research, which is covered by the *core-set* of dental journals. The major goal of science is to respond to the needs and challenges of society, by creating relevant knowledge that brings benefit to society. Hence, research policy makers within research institutes and on the national level, as well as research funders, hold an important responsibility for the focus of research activities [40].

The increasing focus on non-dental research fields may result in a decline in research serving the dental healthcare services (dental healthcare professionals and patients). We would argue that a balanced dental research portfolio is of importance for both policy in science and dental care. Hence, when designing policy interventions in the research system, research policy makers should reflect on whether this will induce changes in the dental research field dynamics that are meeting their goals: will the interventions serve to stimulate research addressing dental care and societal challenges in the oral healthcare field, or are they – unintended - stimulating further academic drift towards basic sciences?

## Conclusion

Our findings suggest that academic drift has been influencing the research agenda in the dental research field. This is reflected in the changing focus of dental research institutes over the last decades towards an increasing share of publications in non-dental basic science journals and in clinical medical journals, and in the fairly limited and constant knowledge streams from basic science to dental research. An important task lies with the dental research community and research policy makers to establish a research portfolio that balances achieving scientific progress with serving dental care.

## Supplementary information

Supplementary Information

## References

[1] Merton RK. *The Sociology of Science: Theoretical and Empirical Investigations*. (University of Chicago Press, 1973).

[2] The PLoS Medicine Editors. (2006). The impact factor game. PLoS Med.

[3] Sandström U, van den Besselaar P (2016). Quantity and/or Quality? The importance of publishing many papers. PLOS ONE.

[4] Rice DB, Raffoul H, Ioannidis JPA, Moher D. Academic criteria for promotion and tenure in biomedical sciences faculties: Cross sectional analysis of international sample of universities. BMJ. m2081 (2020). 10.1136/bmj.m2081. 10.1136/bmj.m2081 PMC7315647 32586791

[5] Adam D (2002). The counting house. Nature.

[6] Hicks D, Wouters P, Waltman L, de Rijcke S, Rafols I (2015). Bibliometrics: The leiden manifesto for research metrics. Nat N.

[7] Benedictus R, Miedema F, Ferguson MWJ (2016). Fewer numbers, better science. Nat N..

[8] About Science in Transition. *Science in transition.* https://scienceintransition.nl/en/about-science-in-transition.

[9] Health Council of the Netherlands. Perspectives on oral health care. (2012).

[10] Harwood J (2010). Understanding academic drift: On the institutional dynamics of higher technical and professional education. Minerva.

[11] Seglen PO (1997). Why the impact factor of journals should not be used for evaluating research. BMJ.

[12] van Eck NJ, Waltman L, van Raan AFJ, Klautz RJM, Peul WC (2013). Citation analysis may severely underestimate the impact of clinical research as compared to basic research. PLoS ONE.

[13] Hansson S, Polk M (2018). Assessing the impact of transdisciplinary research: The usefulness of relevance, credibility, and legitimacy for understanding the link between process and impact. Res Eval.

[14] Belcher BM, Rasmussen KE, Kemshaw MR, Zornes DA (2016). Defining and assessing research quality in a transdisciplinary context. Res Eval.

[15] van Drooge L, van den Besselaar P, Elsen GMF, de Haas M, van den Heuvel JJ, Maassen van den Brink H, van der Meulen B, et al. Evaluating the societal relevance of academic research: A guide. ERiC-Evaluating Research in Context (2010).

[16] Vugteveen P, Lenders R, Van den Besselaar P (2014). The dynamics of interdisciplinary research fields: The case of river research. Scientometrics.

[17] Besselaar Pvanden, Leydesdorff L (1996). Mapping change in scientific specialties: A scientometric reconstruction of the development of artificial intelligence. J Am Soc Inf Sci..

[18] Porter AL, Rafols I (2009). Is science becoming more interdisciplinary? Measuring and mapping six research fields over time. Scientometrics.

[19] Sanz E, Aragon I, Mendez A (1995). The function of national journals in disseminating applied science. J Inf Sci..

[20] Sambunjak D (2009). National vs. international journals: Views of medical professionals in Croatia. Learn Publ..

[21] Bollen J, Rodriquez MA, Van de Sompel H (2006). Journal status. Scientometrics.

[22] Vélez-Cuartas G, Lucio-Arias D, Leydesdorff L (2016). Regional and global science: Publications from Latin America and the Caribbean in the SciELO citation index and the Web of Science. Prof Inf..

[23] Leydesdorff L, Bihui J (2005). Mapping the Chinese science citation database in terms of aggregated journal–journal citation relations. J Am Soc Inf Sci Technol..

[24] Noyons E (2019). Measuring societal impact is as complex as ABC. J Data Inf Sci.

[25] 2025-Vision for Science. Ministry of Education, Culture and Science. The Hague, The Netherlands (2014).

[26] Haslam N, Lusher D (2011). The structure of mental health research: Networks of influence among psychiatry and clinical psychology journals. Psychol Med..

[27] European Commission. *A public-private partnership on the future internet: Communication from the Commission to the European Parliament, the Council, the European Economic and Social Committee and the Committee of the Regions*. (EUR-OP, 2009).

[28] de Solla Price DJ. *Little science, big science-- and beyond*. (Columbia University Press, 1986).

[29] Yeung A, Leung W (2018). Citation network analysis of dental implant literature from 2007 to 2016. Int J Oral Maxillofac Implants.

[30] Tarazona-Alvarez B, Lucas-Dominguez R, Paredes-Gallardo V, Alonso-Arroyo A, Vidal-Infer A (2019). A bibliometric analysis of scientific production in the field of lingual orthodontics. Head Face Med.

[31] Zhang Q, Yue Y, Shi B, Yuan Z (2018). A Bibliometric analysis of cleft lip and palate-related publication trends from 2000 to 2017: *Cleft Palate*. Craniofac J..

[32] Ahmad P, Asif JA, Alam MK, Slots J (2020). A bibliometric analysis of Periodontology 2000. Periodontol 2000.

[33] Ahmad P, Alam MK, Jakubovics NS, Schwendicke F, Asif JA (2019). 100 years of the *Journal of Dental Research*: A bibliometric analysis. J Dent Res..

[34] Hilário CM, Grácio MCC (2017). Scientific collaboration in Brazilian researches: A comparative study in the information science, mathematics and dentistry fields. Scientometrics.

[35] De la Flor-Martínez M (2017). Evaluation of scientific output in dentistry in Spanish Universities. Med Oral Patol Oral Cir Bucal.

[36] Gil-Montoya JA, Navarrete-Cortes J, Pulgar R, Santa S, Moya-Anegon F (2006). World dental research production: An ISI database approach (1999-2003). Eur J Oral Sci..

[37] Pulgar R, Jiménez-Fernández I, Jiménez-Contreras E, Torres-Salinas D, Lucena-Martín C (2013). Trends in world dental research: An overview of the last three decades using the Web of Science. Clin Oral Investig..

[38] Skvoretz J (2016). Research and practice communications between oral health providers and prenatal health providers: A bibliometric analysis. Matern Child Health J.

[39] Bornmann L, Stefaner M, de Moya Anegón F, Mutz R (2014). What is the effect of country-specific characteristics on the research performance of scientific institutions? Using multi-level statistical models to rank and map universities and research-focused institutions worldwide. J Informetr..

[40] Chalmers I (2014). How to increase value and reduce waste when research priorities are set. Lancet.

