# Supplementary material for: Evidence and consequences of academic drift in the field of dental research: A bibliometric analysis 2000–2015
Source: BDJ Open. 2022 Jan 17;8:3. doi: 10.1038/s41405-022-00093-w (PMC8763897; doi:10.1038/s41405-022-00093-w)
Supplement: Supplementary file 1 — Supplementary Information [file 41405_2022_93_MOESM1_ESM.docx]

**Supplementary Files**

**Annex-A**: **Selecting the entrance journals.**

For each of the three years, the journals from the WoS category Dentistry with the highest impact factor were selected. The number of journals used as entrance journals depended on the number of journals that were related through citation relations to these entrance journals. This number was set at a maximum number of 250 journals in the map in order to keep the visualization readable. For 2000 and 2015, the number of entrance journals was 26, for 2008, it was 27 journals.

**Supplementary Table 1. Entrance journals per year**

| 2000 (26 journals) | 2008 (27 journals) | 2015 (26 journals) |
| --- | --- | --- |
|  | Acta Odontol Scand |  |
|  | Am J Dent |  |
| Am J Orthod Dentofac | Am J Orthod Dentofac | Am J Orthod Dentofac |
| Angle Orthod | Angle Orthod | Angle Orthod |
| Arch Oral Biol | Arch Oral Biol | Arch Oral Biol |
| Brit Dent J | Brit Dent J | Brit Dent J |
| Caries Res | Caries Res | Caries Res |
| Cleft Palate-Cran J | Cleft Palate-Cran J | Cleft Palate-Cran J |
| Clin Oral Implan Res | Clin Oral Implan Res | Clin Oral Implan Res |
| Community Dent Oral | Community Dent Oral | Community Dent Oral |
| Dent Mater | Dent Mater | Dent Mater |
| Int Endod J |  | Int Endod J |
| Int J Oral Max Impl | Int J Oral Max Impl | Int J Oral Max Impl |
|  | Int J Periodont Rest |  |
| Int J Oral Max Surg |  | Int J Oral Max Surg |
| J Am Dent Assoc | J Am Dent Assoc | J Am Dent Assoc |
| J Clin Periodontol | J Clin Periodontol | J Clin Periodontol |
| J Cranio Maxill Surg |  | J Cranio Maxill Surg |
| J Dent | J Dent | J Dent |
| J Dent Res | J Dent Res | J Dent Res |
| J Endodont | J Endodont | J Endodont |
| J Oral Maxil Surg | J Oral Maxil Surg | J Oral Maxil Surg |
| J Oral Pathol Med | J Oral Pathol Med | J Oral Pathol Med |
| J Oral Rehabil | J Oral Rehabil | J Oral Rehabil |
| J Periodontal Res | J Periodontal Res | J Periodontal Res |
| J Periodontol | J Periodontol | J Periodontol |
| J Prosthet Dent | J Prosthet Dent | J Prosthet Dent |
| Oral Microbiol Immun | Oral Microbiol Immun |  |
| Oral Oncol | Oral Oncol | Oral Oncol |
|  | Oral Surg Oral Med O | Oral Surg Oral Med O |

**Annex-B: Selecting the countries**

For between-country comparison we focused on the countries with the largest volume of publications in the most important journals (in terms of impact factor) of the dental research field from 1998 up to 2015. To overcome the differences per year in the list of top-10 impact factor journals we identified all journals that have been listed in the top-10 from 1998 up to 2016. For this we used the Journal Citation Reports for journal impact factors from 1998 up to 2016 for the category *Dentistry, Oral Surgery & Medicine*. The resulting 30 journals were ranked by their multiple-year impact factor, and the ten highest ranked, i.e. multiple-year top-10, were included for further analysis (table below).

**Supplementary Table 2:** Ranking list of Top-10 Impact Factor Dental Journals between 1998 and 2016 based on rank sum.

|  | **Journal Title** | **Ranksum** | **Average IF** | **No. times in IF top-10** | **No. years included since 1997** |
| --- | --- | --- | --- | --- | --- |
| 1 | Journal of Dental Research | 186 | 3,707 | 20 | 20 |
| 2 | Periodontology 2000 | 122 | 2,738 | 16 | 20 |
| 3 | Journal of Clinical Periodontology | 114 | 2,652 | 19 | 20 |
| 4 | Dental Materials | 101 | 2,557 | 17 | 20 |
| 5 | Clinical Oral Implants Research | 95 | 2,507 | 17 | 20 |
| 6 | Critical Reviews in Oral Biology Med. | 92 | 3,300 | 10 | 10 |
| 7 | Oral Oncology | 88 | 2,425 | 16 | 20 |
| 8 | Journal of Endodontics | 46 | 2,099 | 10 | 20 |
| 9 | Clinical Implant Dentistry and Related Research | 39 | 3,027 | 8 | 10 |
| 10 | Journal of Periodontology | 36 | 2,102 | 8 | 20 |

To compose the ranking list, points were assigned to each position in the top 10 impact factor dental journals as follows: position 1=10 points, 2=9 points, 3=8 points, 4=7 points, 5=6 points, 6=5 points, 7=4 points, 8=3 points, 9=2 points, 10=1 point. The sum of assigned points is found in column 3.

We identified the ten countries that produced the most publications to the multiple-year top-10 journals. Seven countries, USA, England, Germany, Italy, The Netherlands, Sweden, Switzerland were used for further analysis. As earlier research has shown that the research portfolio of upcoming countries is different from those of the more established countries (Horlings & Van den Besselaar 2011), Brazil and China, as the upcoming countries during the studied period, were excluded from analysis. Japan was excluded from analysis as earlier research this country shows very different changes in the research portfolio (Van den Besselaar, Heyman, Sandstrom 2018).

**ANNEX-C: Identifying local journals**

Journals were classified as local when its title included a country or a continent. Journals including British, American, Australian or New Zealand in their journal titles are not exclusively local because they are published in the English language. Therefore, we calculated the correlation between the countries share in the *core-set* of dental journals and the countries share in journals that might be identified as local. If this correlation was less than 0.5 the journal was identified as local.

The Supplementary Table 3 below shows the journals that according to that criterion would be classified as local. For each of these journals we calculated which country dominates the journal (and in some cases which two countries dominate the journal). The second column shows that country, and the percentage of papers in the journals that are have an author of that country. For example, in *Acta Odontologica Scandinavica*, Norway and Sweden are good for more than 40% of all papers, which is much more than would be expected based on the share of those countries in the total world production of dental research papers. This the case for all journals in the list. As a second check, we show the share of the US publications in the journals, the largest producer of dental research papers. In the local US journals, the share of the US is much higher than expected, whereas in all other local journals, the share of the US is much lower than expected. Overall this suggests that our definition of local/national journals seems to be correct.

| **Journal**  Acta Odontologica Scandinavica  Australian Dental Journal  Australian Endodontic Journal  Australian Orthodontic Journal  Brazilian Oral Research  British Dental Journal  British Journal Of Oral Maxillofacial Surgery  European Journal Of Dental Education  European Journal Of Oral Implantology  European Journal Of Orthodontics  European Journal Of Paediatric Dentistry  Journal Of Orofacial Orthopedics Fortschritte Der Kieferorthopadie  Journal Of The American Dental Association  Journal Of The Canadian Dental Association  Korean Journal Of Orthodontics  Medicina Oral Patologia Oral Y Cirugia Bucal  Oral And Maxillofacial Surgery Clinics Of North America  Revue De Stomatologie De Chirurgie Maxillo Faciale Et De Chirurgie Orale  Revue De Stomatologie Et De Chirurgie Maxillo Faciale  Swedish Dental Journal | **% Dominant**  30% (Swe), 13% (Norw)  70% (Australia)  23% (Brazil), 17% (Aus)  21% (Aus), 14% (Swe)  91% (Brasil)  83% (UK)  51% (UK)  27% (UK), 13% (NL)  55% (It), 43% (Swe)  15% (UK), 11% (Turkey)  47% (Italy)  87% (Germany)  83% (US)  58% (Canada)  7% (Korea)  53% (Spain)  90% (US)  70% (France)  75% (France)  94% (Sweden) | **% US**  7%  4%  7%  8%  4%  3%  13%  4%  6%  3%  3%  83%  14%  9%  3%  90%  1%  1%  2% |
| --- | --- | --- |

**Supplementary Table 3: Local journals**

**Annex-D**: **Clusters of journals in the field of dental research in 2008 - results of the factor analysis**

**Supplementary Table 4**

| **0 General dentistry** | **3. Public health / general medicine** | **7. Biochemistry** |
| --- | --- | --- |
| j dent | j public health-uk | j biol chem |
| am j dent | bmc public health | j cell physiol |
| eur j oral sci | brit med j | j mol histol |
| j dent res | public health | front biosci |
| j am dent assoc | cad saude publica | Fluoride |
| int dent j | cochrane db syst rev | cell tissue res |
| j can dent assoc | cienc saude coletiva | Carcinogenesis |
| brit dent j | health policy | biotechnol biotec eq |
| j dent sci | lancet | j physiol pharmacol |
| oral dis | arch intern med | **8 Orthodontics** |
| swed dent j | j altern complem med | am j orthod dentofac |
| **1 Oncology** | int j lang comm dis | aust orthod j |
| j cancer res clin | int j circumpol heal | eur j orthodont |
| oncol rep | croat med j | angle orthod |
| anticancer res | j eur acad dermatol | j orofac orthop |
| brit j cancer | **4 Implantology** | korean j orthod |
| hum cell | implantologie | orthod craniofac res |
| int j cancer | int j oral max impl | **9 Microbiology** |
| expert rev anticanc | clin oral implan res | adv appl microbiol |
| clin cancer res | implant dent | fems microbiol let |
| int j oncol | clin implant dent r | appl environ microb |
| cancer sci | iti treatment guide | can j microbiol |
| cancer | int j periodont rest | microbiol-sgm |
| j surg oncol | periodontol 2000 | Anaerobe |
| cancer epidem biomar | int j med robot comp | bmc microbiol |
| oral oncol | **5. Community dentistry** | j bacterial |
| in vivo | aust dent j | **10 Oral surgery** |
| eur j cancer care | community dent oral | brit j oral max surg |
| j int med res | community dent hlth | j oral maxil surg |
| j cancer educ | int j paediatr dent | oral radiol |
|  | j public health dent | med oral patol oral |
| **2 Operative dentistry & materials** | j dent educ | int j oral max surg |
| j adhes dent | j clin pediatr dent | oral surg oral med o |
| oper dent | eur j dent educ | rev stomatol chir |
| dent mater j | acta odontol scand | dentomaxillofac rad |
| dent mater | pediatr dent | j cranio maxill surg |
| j esthet restor dent | caries res | j oral pathol med |
| j appl oral sci | **6 Plastic surgery** | **11. Biomaterials** |
| quintessence int | plast reconstr surg | acta biomater |
| mater res-ibero-am j | j plast reconstr aes | j biomed mater res a |
| j prosthet dent | ann chir plast esth | j mater sci-mater m |
| int j prosthodont | clin plast surg | Biomaterials |
| clin oral invest | scand j plast recons | key eng mater |
| gerodontology | facial plast surg | j biomed mater res b |
| arch oral biol | j craniofac surg |  |
|  | acta cir bras |  |
|  | cleft palate-cran j |  |
|  | clin anat |  |

**Supplementary Table 4 – continued**

| **12 Clinical microbiology** | **18 Neuroscience** | **26 Public health (USA)** |
| --- | --- | --- |
| new microbiol | j neurophysiol | am j public health |
| j clin microbiol | j neurosci | health place |
| med mycol | neuroscience | public health rep |
| j med microbiol | exp brain res | **27 Genetics** |
| biomedica | clin neurophysiol | am j med genet a |
| clin infect dis | **19 Immunology** | eur j med genet |
| braz j microbiol | infect immun | birth defects res a |
| antimicrob agents ch | microb pathogenesis | **28 Chemistry** |
| curr hiv res | fems immunol med mic | p soc photo-opt ins |
| **13 Pain** | oral microbiol immun | j phys chem b |
| eur j pain | j immunol | chem rev |
| j pain | **20 General medicine** | j phys d appl phys |
| clin j pain | clinics | j appl polym sci |
| pain | new engl j med | **29 Geriatrics** |
| j musculoskelet pain | expert opin pharmaco | j am geriatr soc |
| j orofac pain | **21 Anatomy** | j gerontol nurs |
| **14 Otorhinolaryngology** | j hum evol | **30 Quality of life** |
| eur arch oto-rhino-l | j anat | qual life res |
| laryngoscope | homo | health qual life out |
| arch otolaryngol | anat rec | **31 Forensic science** |
| int j pediatr otorhi | anthropol sci | forensic sci int |
| curr opin otolaryngo | integr comp biol | j forensic sci |
| head neck-j sci spec | j archaeol sci | **32 Radiation** |
| hno | **22 TMD** | radiat prot dosim |
| **15 Endodontology** | cranio | health phys |
| aust endod j | j oral rehabil | rofo-fortschr rontg |
| j endodont | appl psychophys biof | **33 Antropology** |
| int endod j | **23 Kinesiology** | collegium antropol |
| **16 Periodontology** | spine | **34 Medical devices** |
| j clin periodontol | eur spine j | expert rev med devic |
| j periodontol | **24 Biomechanics** |  |
| j periodontal res | rev bras fisioter |  |
| odontology | j electromyogr kines |  |
| **17 Pediatrics** | j biomech |  |
| pediatrics | ann biomed eng |  |
| acta paediatr | int j sports med |  |
| pediatr emerg care | bone |  |
| dev disabil res rev | **25 Laser** |  |
| pediatr pulm | laser surg med |  |
| j pediatr surg | photomed laser surg |  |
| pediatr anesth | laser med sci |  |
|  | j biomed opt |  |

**Annex-E: Distribution of dental research institutes output over WoS categories**

**Supplementary Table 5**

| **Discipline** | **Change*** | **Average share 98-00** | **Average**  **share 14-15** | **Category**** |
| --- | --- | --- | --- | --- |
| Biomaterials / materials | up | 4,8% | 5,5% | b |
| Multidisciplinary sciences | up | 1,2% | 4,9% | o |
| Biochemistry molecular biology | up | 3,2% | 3,9% | b |
| Cell biology | up | 2,6% | 3,7% | b |
| Genetics heredity | up | 2,1% | 3,3% | b |
| Medicine research experimental | up | 0,6% | 3,0% | b |
| Clinical neurology | up | 1,1% | 2,7% | c |
| Medicine general internal | up | 1,3% | 2,6% | c |
| Endocrinology metabolism | up | 1,6% | 2,4% | b |
| Public environmental occupational health | up | 2,0% | 2,3% | p |
| Chemistry | up | 0,9% | 1,6% | b |
| Pediatrics | up | 1,2% | 1,5% | c |
| Health care / policy sciences | up | 0,4% | 1,4% | p |
| Surgery | equal | 7,6% | 7,8% | c |
| Engineering biomedical, tissue, ect | equal | 7,1% | 7,5% | b |
| Neurosciences | equal | 2,7% | 2,9% | b |
| Otorhinolaryngology | equal | 1,2% | 1,4% | c |
| Biochemical research methods | equal | 0,7% | 0,7% | b |
| Dentistry oral surgery medicine | down | 60,5% | 42,6% | d |
| Oncology | down | 4,3% | 3,1% | c |
| Immunology | down | 3,1% | 2,4% | b |
| Microbiology | down | 2,5% | 2,2% | b |
| Pharmacology pharmacy | down | 2,1% | 1,7% | o |
| Radiology nuclear medicine medical imaging | down | 1,5% | 1,3% | o |
| Pathology | down | 1,8% | 0,9% | c |
| Anatomy morphology | down | 1,1% | 0,4% | b |

* change is based on the change in average share. Changes lower than 10% are considered as ‘equal’.

** b= basic research, c= clinical research, d= dental research, o= other, p= public health & policy
